# Association between post-procedural hyperoxia and poor functional outcome after mechanical thrombectomy for ischemic stroke: an observational study

**DOI:** 10.1186/s13613-019-0533-8

**Published:** 2019-05-24

**Authors:** Héctor Vargas López, Miguel Fernández Vivas, Rafael Núñez Ruiz, José Ros Martínez, Blanca García-Villalba Navaridas, Manuel García Villa, Cristina Llamas Lázaro, Rubén Jara Rubio, Ana Morales Ortiz, Laura Albert Lacal, Antonio Moreno Diéguez

**Affiliations:** 1 Department of Intensive Care, Virgen de la Arrixaca, Clinical University Hospital, Murcia, Spain; 2 Department of Neuroradiology, Virgen de la Arrixaca, Clinical University Hospital, Murcia, Spain; 3 Department of Neurology, Biomedical Research Institute of Murcia (IMIB), Virgen de la Arrixaca, Clinical University Hospital, Murcia, Spain

**Keywords:** Hyperoxia, Stroke, Mortality, Ischemic, Oxygen, Cerebral blood flow

## Abstract

**Background:**

The actual effects of oxygen therapy on patients who have suffered a stroke are still unknown, and its recommendation as a routine measure in emergency services remains controversial. The aim of this study is to determine the effect of hyperoxia in functional recovery in patients with ischemic stroke who underwent intra-arterial mechanical thrombectomy (IAMT).

**Methods:**

A prospective observational cohort study that included all adult patients consecutively admitted to the intensive care unit (ICU) due to an ischemic stroke in the anterior cerebral circulation and following an IAMT intervention, between 2010 and 2015. All patients were intubated and connected to mechanical ventilation for the intra-arterial therapy, receiving supplementary oxygen to achieve saturations above 94%. Two groups were established regarding oxygen partial pressure (paO_2_) reached. It was based on a single ICU admission blood gas analysis. The hyperoxia group was defined as paO_2_ > 120 mmHg. We measured functional recovery in each of the groups according to the modified Rankin scale after 90 days.

**Results:**

For the analysis, a total of 333 patients were included. High levels of paO_2_ were mostly related to higher scores in modified Rankin scale (mRS) after 90 days. There were 60.6% cases with mRS ≥ 4 and 70.6% with mRS ≥ 3 in the hyperoxia group, compared to 43.0% and 56.1% in the paO_2_ ≤ 120 group, *p* < 0.01, respectively. Mortality was higher in the hyperoxia group, 28.6% vs 18.7%, *p* = 0.04. After regression adjustment by confounding factors, poor functional outcome was still significantly higher in the hyperoxia group, for both mRS ≥ 4 and mRS ≥ 3: OR 2.2.7, IC 95%, 1.22–4.23, *p* = 0.01 and OR 2.07, IC 95%, 1.05–4.029, *p* = 0.04, respectively. Both the National Institute of Health Stroke Scale Score (NIHSS) values at 24 h after the IAMT and the days of ICU stay were significantly higher in the hyperoxia group.

**Conclusions:**

In patients with ischemic stroke in the anterior cerebral circulation treated with IAMT, we found an association between admission PaO2 > 120 mmHg and worse functional outcome 90 days after ischemic stroke, but this association needs further confirmation by other studies.

**Electronic supplementary material:**

The online version of this article (10.1186/s13613-019-0533-8) contains supplementary material, which is available to authorized users.

## Background

According to the World Health Organization’s data analysis, cerebrovascular diseases are the third leading cause of death in the Western world and the first cause of physical disability in adults. Over 15 million people per year, the equivalent of one in 400 people, suffer from a stroke in the world [1].

In Spain, the overall rate of ischemic stroke, excluding transient ischemic attacks, is 140 per every 100,000 inhabitants per year [2].

The brain is an extremely sensitive organ to hypoxia. Insufficient oxygen supply leads to a critical cerebral metabolic situation. Hypoxia during stroke has been related to a higher death risk [3], since it can worsen the lesions resulting from cerebral ischemia in this kind of patients, especially in those patients whose cerebral flow autoregulation mechanism has been affected by ischemia.

Until a few years ago, the only reperfusion therapy approved for the treatment of patients with stroke was intravenous thrombolysis by tissue plasminogen activators, such as alteplase, within the first 4.5 h after the stroke.

Recently, and particularly after the publication of the results obtained in the “MrClean” multicenter trial [4], it has been proved that endovascular recanalization therapies by means of mechanical devices are faster and have a higher success rate in vessel revascularization, especially in strokes affecting large proximal arteries [5, 6], and they also allow widening the therapeutic window up to 8 h in the case of anterior territory strokes [7].

Although oxygen therapy is frequently used to minimize the effects of hypoxia in patients who have suffered a stroke, its role is still controversial and it is not without problems [8]. Oxygen therapy can worsen the ventilation–perfusion ratio and cause atelectasis or cerebral, coronary and systemic vasoconstriction.

Oxidative stress arising from the production of reactive oxygen species has been proposed as a basic cause of brain damage in cerebrovascular accidents particularly during the reperfusion subsequent to revascularization of the affected territory [9, 10].

In the medical literature, there is no consensus regarding the real effects of oxygen therapy in patients who have suffered a stroke, or regarding its recommendation as a routine measure in emergency services. There are studies focused on its possible side effects [11], while others defend its probable neuroprotective role in allowing the widening of the narrow therapeutic window until thrombolysis/thrombectomy is performed [12].

This study evaluates the role of oxygen supplementation in patients with ischemic stroke.

## Methods

### Aims of the study

The main goal of this study was to determine the effect of hyperoxia in functional recovery in patients with ischemic stroke who underwent intra-arterial mechanical thrombectomy (IAMT).

### Design and setting

This was a prospective observational cohort study, with follow-up without repeated measures.

The study was approved by the Hospital Ethics Committee, and since there was no interference with patients’ management, signed informed consent was waived.

The study population included all patients consecutively admitted to the intensive care unit (ICU) of the Virgen de la Arrixaca Clinical University Hospital, Murcia (Spain), because of an ischemic stroke in the anterior cerebral circulation and following an IAMT, between April 2010 and December 2015.

The relationship between the oxygenation of the patients and the obtaining of a poor functional outcome was evaluated 90 days after the intervention.

The baseline neurological status of the patient, modified Rankin scale (mRS), National Institute of Health Stroke Scale Score (NIHSS) and Glasgow Coma Scale (GCS) were registered at the time of arrival at hospital. An urgent blood test, electrocardiogram and neuroimaging protocol with multimodal CT (unenhanced *CT*, *perfusion CT* and CT angiography) were performed. O_2_ was administered to maintain arterial saturation over 94%.

After diagnosis of ischemic stroke and location of the responsible vessel occlusion using imaging techniques, a fibrinolysis with alteplase (rtPA) was performed on patients who were within a period of < 4.5 h since the onset of symptoms and who met the selection criteria (Additional file 1). In the cases in which these criteria were not met, or those in which the patient’s evolution was not favorable, intra-arterial mechanical thrombectomy was considered by the Neuroradiology Service. It was performed on patients who had an obstruction of the intracranial or extracranial internal carotid artery or of the trunk of the middle cerebral artery, with a mismatch of at least 30% by CT perfusion, when no more than 8 h had elapsed from the onset of symptoms, as long as there were no contraindications (Additional file 1).

All patients for the intra-arterial therapy were intubated and connected to mechanical ventilation. They received supplementary oxygen to achieve saturations above 94%, according to the criteria of the anesthesiologists. After the intervention, they were admitted in ICU. FiO2 and PEEP levels delivered at ICU admission: 0.5 (0.4–0.6) and 6 (5–7), respectively (variables expressed as median and interquartile range). Some patients were extubated prior to ICU admission. These patients received supplementary oxygen: FO2 0.5 (0.5–0.8), variable expressed as median and interquartile range.

Once in the unit, arterial oxygen partial pressure (paO_2_) was measured by means of a control blood gas analysis, according to usual clinical practice. Two groups were formed. They were based on the paO2 levels reached of a single ICU admission blood gas analysis. As a cutoff, a paO2 of 120 mmHg was established, according to the literature review [13]. The hyperoxia group was defined as paO_2_ > 120 mmHg.

We evaluated the degree of functional recovery in each of the groups after 90 days (primary endpoint), objectively measured according to the mRS through a structured interview. This scale has 7 scores, which range from 0 (no symptoms at all) to 6 (dead).

The length of the patient’s stay in the ICU and the NIHSS were included as secondary endpoints after the procedure for each study group.

They stayed in this unit for at least 24 h, depending on their progress. Then, they were admitted into the intermediate care unit specially prepared for that purpose.

### Statistical analysis

A univariate analysis was performed in order to study the relationship between hyperoxia with baseline characteristics and the variables established as primary and secondary endpoints. We used the Chi-squared test for qualitative variables and the Mann–Whitney *U* test for quantitative variables.

We performed a statistical adjustment by logistic regression to study the relationship between hyperoxia and a poor functional outcome. The variables that met criteria of confounding factors were introduced in the model. The model included variables that were significant at *p* value of less than 0.25 in univariate analysis. Finally, we obtained the most parsimonious model that did not modify the odds ratio in more than 10% [14]. The results were presented as an OR between hyperoxia and mRS ≥ 4 and mRS ≥ 3 at 90 days, adjusted and unadjusted, with a 95% confidence interval.

## Results

**Fig. 1:**
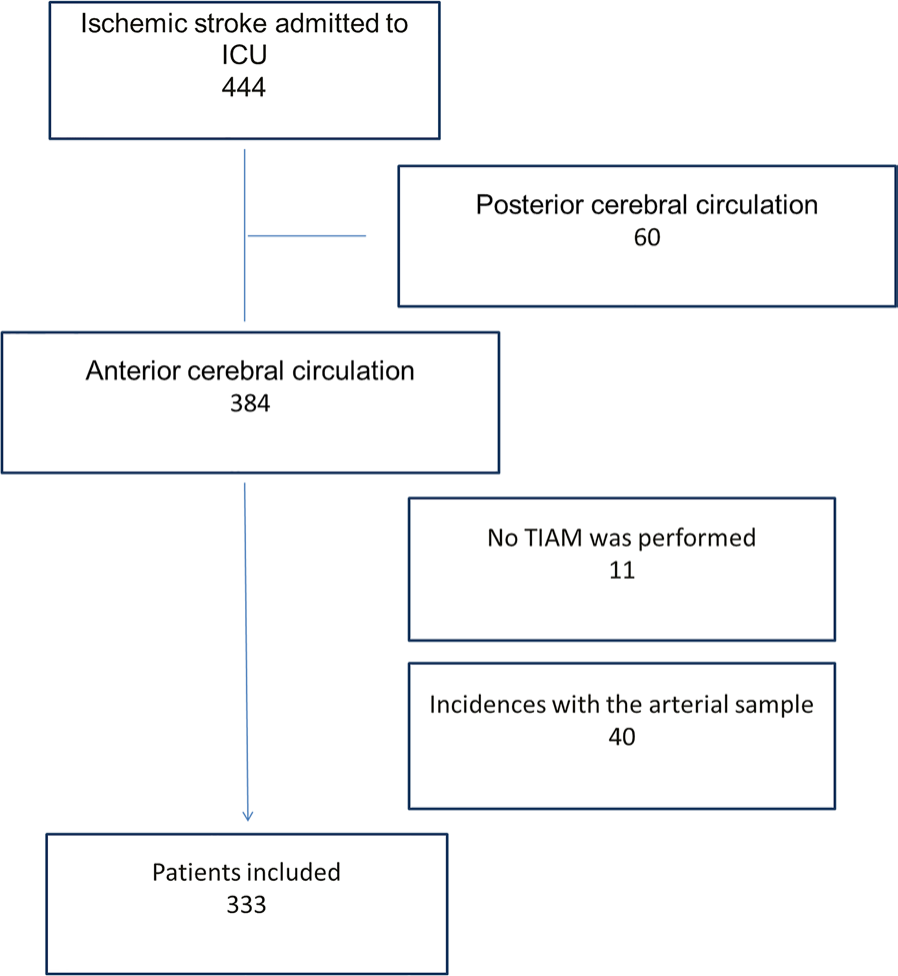
Flowchart of patients included in the study

During the study period, 444 patients with a diagnosis of ischemic stroke were admitted to the unit, of whom 384 corresponded to an anterior cerebral circulation due to large vessel occlusion. Of these, 11 did not undergo thrombectomy: 1 complete reperfusion at the time of thrombectomy as a consequence of previous fibrinolysis; 8 due to technical impossibility; 2 because they were cases of carotid dissection that required permanent stent implantation without thrombectomy. Of the 373 resulting, in 40, arterial gasometry was not available due to incidents in the pre-analytic phase at ICU admission, so a total of 333 patients were finally included in the study (Fig. 1).

**Fig. 2:**
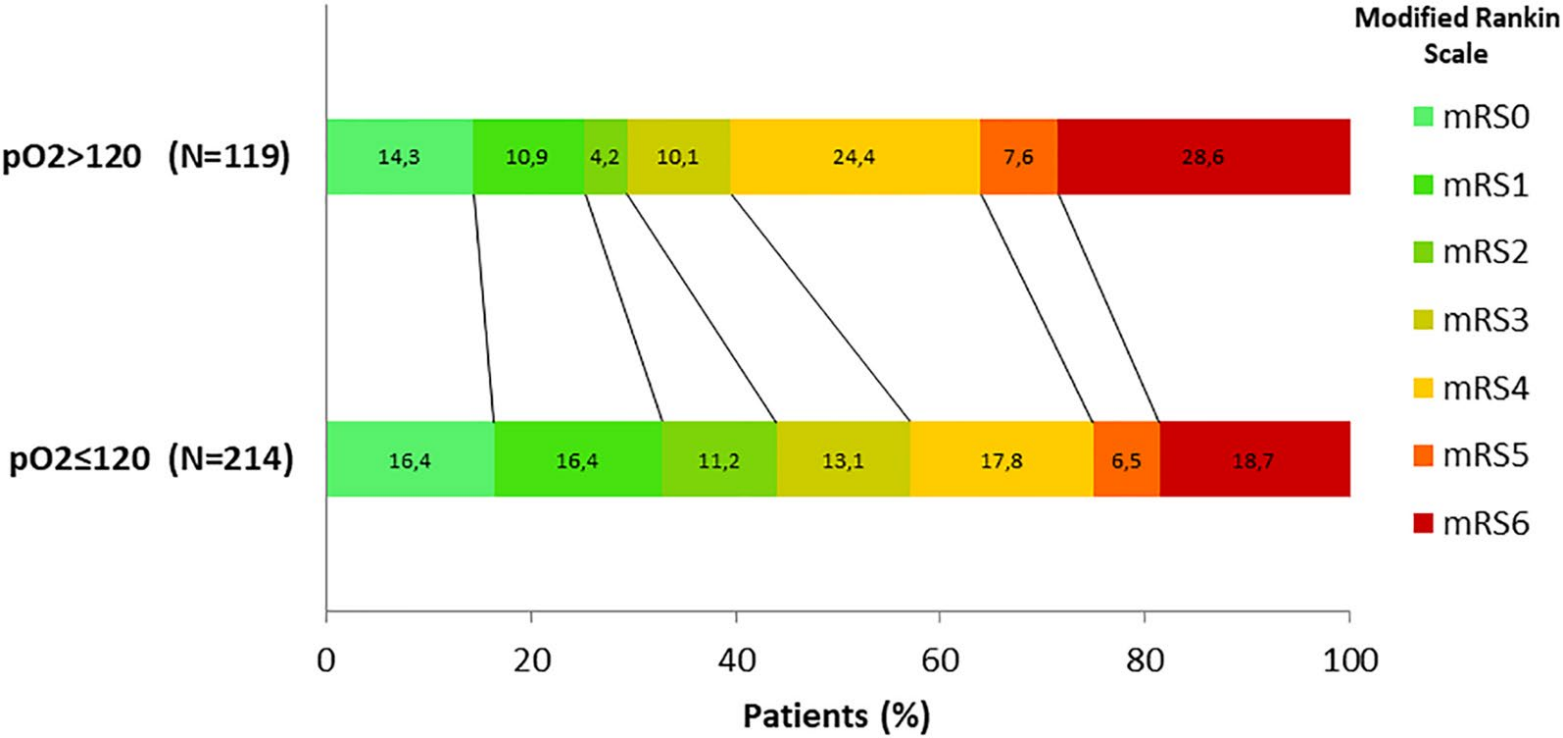
Distribution of scores in the modified Rankin scale at 90 days after IAMT for each group. Score distribution in both groups was unequal, in a significant way: 60.6% of cases with mRS ≥ 4 and 70.6% with mRS ≥ 3 were observed in the hyperoxia group, in comparison with 43.0% and 56.1%, respectively, observed in paO2 < 120 group, *p* < 0.01. Mortality was higher in the hyperoxia group, 28.6% vs 18.7%, *p* = 0.04

The score distribution on the modified Rankin scale 90 days after IAMT between the two groups was clearly unequal. Thus, 60.6% of cases with mRS ≥ 4 and 70.6% with mRS ≥ 3 were observed in the hyperoxia group, compared to 43.0% and 56.1% in the paO_2_ ≤ 120 group, OR 2.03 (CI 95%, 1.29–3.21, *p* < 0.01) and 2.03 (CI 95%, 1.29–3.21, *p* < 0.01), respectively. Thus, low levels of paO_2_ were mostly linked with lower mRS scores (Fig. 2).

Significant differences regarding the baseline situation of patients were observed (Table 1).

**Table 1:** Characteristics of patients at ICU admission

Characteristic	pO2 ≤ 120 mmHg *N* = 214	pO2 > 120 mmHg *N* = 119	Significance level (*p* value)
Age in years	71 (60–78)	71 (57–77)	0.49
Male sex	107 (50.0)	55 (46.2)	0.51
Body mass index	27 (25–30)	26 (24–28)	0.02
History of smoking	66 (30.8)	31 (26.1)	0.36
Baseline Rankin			0.95
0	151 (70.9)	86 (72.3)	0.79
1	44 (20.7)	19 (16.0)	0.30
≥ 2	18 (8.5)	14 (11.8)	0.33
Hypertension	150 (70.1)	75 (63.0)	0.19
Dyslipidemia	96 (44.9)	48 (40.3)	0.42
Diabetes mellitus	61 (28.5)	34 (28.6)	0.99
History of COPD^a^	19 (8.9)	7 (5.9)	0.33
History of CRF^b^	12 (5.6)	7 (5.9)	0.92
Atrial fibrillation/flutter	88 (41.1)	45 (37.8)	0.55
Previous stroke	26 (12.1)	12 (10.1)	0.57
Previous antiaggregating therapy	64 (29.9)	39 (32.8)	0.59
Previous anticoagulation therapy	35 (16.4)	18 (15.1)	0.77
Preprocedure GCS	14 (13–15)	14 (12–15)	0.44
Preprocedure NIHSS	18 (12–21)	19 (14–22)	0.09
APACHE	15 (12–19)	16 (12–22)	0.23
Findings in CT scan			0.11
Without ischemia	80 (38.1)	53 (44.5)	0.25
Acute ischemic areas^c^	103 (49.0)	59 (49.6)	0.93
Areas of ancient stroke	27 (12.9)	7 (5.9)	0.05
Mismatch	70 (50–80)	70 (50–80)	0.43
Etiology of stroke			0.01
Cardioembolic	96 (44.9)	34 (28.6)	< 0.01
Atherothrombotic	69 (32.2)	48 (40.3)	0.14
Idiopathic	49 (22.9)	37 (31.1)	0.10
Wake-up stroke	25 (11.7)	12 (10.1)	0.66
Time (min.) onset of symptoms—IAMT	270 (210–367)	300 (220–398)	0.24
Type of intervention			0.85
Thrombectomy	180 (84.1)	101 (84.9)	
Thrombectomy + stent	34 (15.9)	18 (15.1)	
Reperfusion degree^e^			0.18
Successful reperfusion	201 (93.9)	107 (89.9)	
Non-successful reperfusion	13 (6.1)	12 (10.1)	
OTI at admission^e^	97 (45.3)	79 (66.4)	< 0.01
MAP^f^	87 (73–98)	90 (77–103)	0.22

This table value of quantitative variables expressed as median and interquartile range. Values of qualitative variables expressed as *n* (%)

^a^Chronic obstructive pulmonary disease

^b^Creatinine clearance < 60 ml/min/1.73 m^2^

^c^Included signs of acute ischemia and indirect signs of ischemia: hyperdense middle cerebral artery sign, hemispheric sulcus and/or insular cortex effacement, contrast attenuation, ventricular compression

^d^Successful reperfusion included TICI 2b and TICI 3. Non-successful reperfusion includes TICI ≤ 2a

^e^Patients intubated at ICU admission after IAMT

^f^Mean arterial pressure at ICU admission after IAMT

Therefore, it was adjusted by logistic regression including the confounding variables identified by a previous univariate analysis (Table 2). Finally, this model was simplified, including only those variables that involved a modification of the OR of the hyperoxia variable greater than 10%, according to the parsimony principle: sex, age, APACHE score, preprocedure NIHSS, wearable tissue area (mismatch), reperfusion degree achieved and persistence of orotracheal intubation at ICU admission (Additional file 2).

**Table 2:** Confounding variables included in the regression model

Characteristic	mRS < 4 *N* = 169	mRS ≥ 4 *N* = 164	Significance level (*p* value)
Age in years	67 (55–76)	74 (66–79)	< 0.01
Male sex	84 (49.7)	78 (47.6)	0.70
History of smoking	57 (33.7)	40 (24.4)	0.06
Baseline Rankin			0.02
0	130 (76.9)	107 (65.6)	0.02
1	27 (16.0)	36 (22.1)	0.16
≥ 2	12 (7.1)	20 (12.3)	0.11
Hypertension	104 (61.5)	121 (73.8)	0.02
Diabetes mellitus	40 (23.7)	55 (33.5)	0.05
History of COPD	10 (5.9)	16 (9.8)	0.19
History of CRF^a^	7 (4.1)	12 (7.3)	0.21
Previous anticoagulation therapy	22 (13.0)	31 (18.9)	0.14
Preprocedure GCS	15 (14–15)	14 (11–15)	< 0.01
Preprocedure NIHSS	16 (11–20)	19 (16–22)	< 0.01
APACHE	13 (12–17)	18 (14–24)	< 0.01
Findings in TC			0.12
Without ischemia	74 (44.3)	59 (36.4)	0.14
Acute ischemic areas^b^	73 (43.7)	89 (54.9)	0.04
Areas of ancient stroke	20 (12.0)	14 (8.6)	0.32
Mismatch	70 (60–80)	60 (40–80)	< 0.01
Wake-up stroke	14 (8.3)	23 (14.0)	0.10
Time (min.) onset of symptoms to IAMT	270 (210–360)	290 (211–390)	0.17
Reperfusion degree^c^			< 0.01
Successful reperfusion	164 (97.0)	144 (87.8)	
Non-successful reperfusion	5 (3.0)	20 (12.2)	
OTI at admission^d^	71 (42.0)	105 (64.0)	< 0.01

This table value of quantitative variables expressed as median and interquartile range. Values of qualitative variables expressed as *n* (%)

^a^Creatinine clearance < 60 ml/min/1.73 m^2^

^b^Included signs of acute ischemia and indirect signs of ischemia: hyperdense middle cerebral artery sign, hemispheric sulcus and/or insular cortex effacement, contrast attenuation, ventricular compression

^c^Successful reperfusion included TICI 2b and TICI 3. Non-successful reperfusion includes TICI ≤ 2a

^d^Patients intubated at ICU admission after IAMT

After adjustment, poor functional outcome remained significantly higher in the hyperoxia group for both mRS ≥ 4 and mRS ≥ 3: OR 2.2.7, IC 95%, 1.22–4.23, *p* = 0.01 and OR 2.07, IC 95%, 1.05–4.029, *p* = 0.04, respectively (Table 3). Thus, hyperoxia is an independent factor of poor functional outcome, being twice as frequent among patients with mRS ≥ 4 or mRS ≥ 3.

**Table 3:** Study outcomes

Score on the mRS at 90 days	paO_2_ ≤ 120 mmHg *N* = 214	paO_2_ > 120 mmHg *N* = 119	Significance level (*p* value)
0–1	70 (32.7)	30 (25.2)	0.15
0–2	94 (43.9)	35 (29.4)	< 0.01
0–3	122 (57.0)	47 (39.5)	< 0.01

Primary endpoint	paO_2_ ≤ 120 mmHg *N* = 214	paO_2_ > 120 mmHg *N* = 119	Unadjusted OR value (CI 95%)	Adjusted OR^a^ value (CI 95%)
mRS ≥ 4 at 90 days	92 (43.0)	72 (60.5)	2.03 (1.29–3.21)	2.27 (1.22–4.23)
mRS ≥ 3 at 90 days	120 (56.1)	84 (70.6)	1.81 (1.12–2.90)	2.07 (1.05–4.09)

Secondary endpoints	paO_2_ ≤ 120 mmHg *N* = 214	paO_2_ > 120 mmHg *N* = 119	Significance level (*p* value)
Days of stay in ICU	2 (2–4)	3 (2–6)	0.03
NIHSS after IAMT^b^	9.5 (3–17)	15 (7–20)	< 0.01

This table value of quantitative variables expressed as median and interquartile range. Values of qualitative variables expressed as *n* (%)

^a^Final model, adjusted for sex, age, APACHE, NIHSS, mismatch, reperfusion level achieved and orotracheal intubation at ICU admission

^b^NIHSS score obtained at 24 h after IAMT

**Fig. 3:**
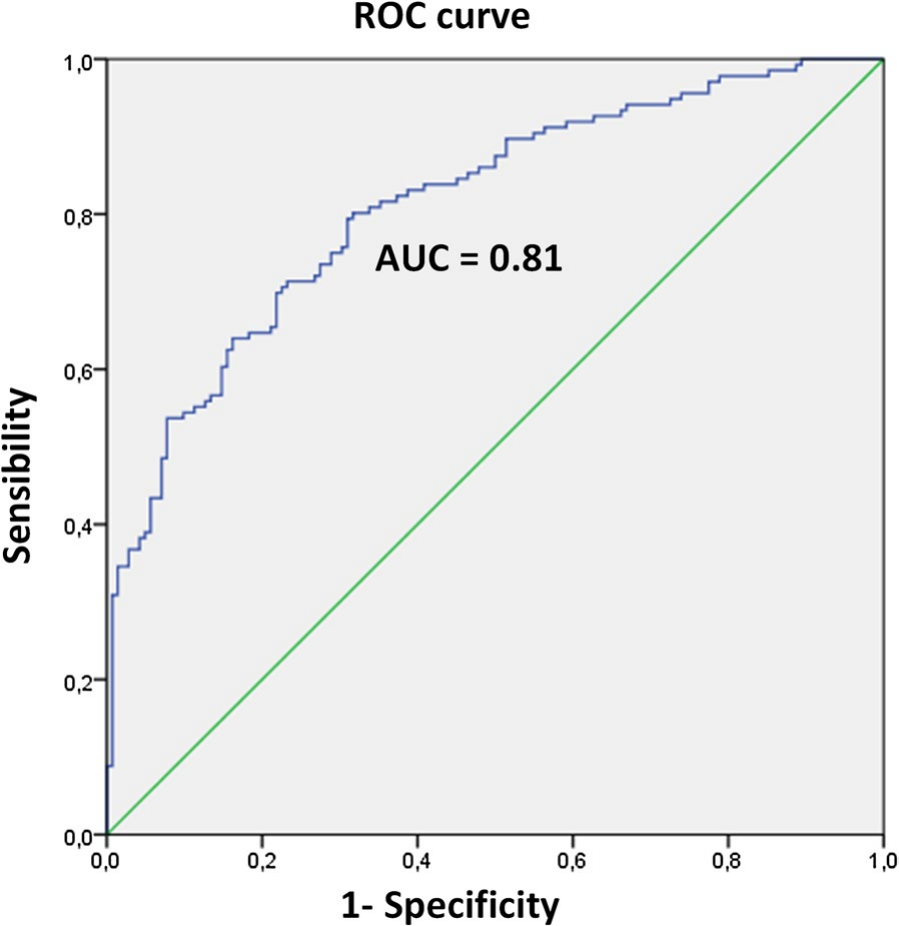
Discriminative capacity of the model. ROC curve

For mRS ≥ 4 outcome, the model obtained showed a sensitivity of 74.6% and a specificity of 71.3%. The discriminative capacity of the model was evaluated through the construction of a ROC curve. An area under the curve of 0.81 was obtained, concluding that in 81% of the cases we will be able to predict the obtaining of a good/poor functional result based on the paO_2_ values (Fig. 3).

On the other hand, both the NIHSS values at 24 h after the IAMT and the days of ICU stay were significantly higher in the hyperoxia group (Table 3).

A subgroup analysis was also performed according to the stroke lesion location (Table 4):

**Table 4:** Subgroup analysis according to the stroke lesion location

Lesion location	pO2 ≤ 120 mmHg *N* = 207	pO2 > 120 mmHg *N* = 115	Significance level (*p* value)
A1^a^	10 (4.7)	7 (5.9)	0.63
M1^b^	125 (58.4)	49 (41.2)	< 0.01
ICA^c^	22 (10.3)	17 (14.3)	0.28
ICA + M1 (tandem lesions)	50 (23.4)	42 (35.3)	0.02

This table value of qualitative variables expressed as *n* (%)

^a^Anterior cerebral artery. A1 segment

^b^Middle cerebral artery. M1 segment

^c^Internal carotid artery

In the subgroup analysis, there were only statistically significant differences for the M1 group. The confounding variables were identified (Additional file 2), and a logistic regression adjusted to these variables was performed. After adjustment, poor functional outcome remained significantly higher in the hyperoxia group for both mRS ≥ 4 and mRS ≥ 3: OR 2.81, IC 95%, 1.12–7.02, *p* = 0.03 and OR 3.56, IC 95%, 1.29–9.84, *p* = 0.01, respectively (Table 5).

**Table 5:** Subgroup analysis

Primary endpoint	paO_2_ ≤ 120 mmHg *N* = 125	paO_2_ > 120 mmHg *N* = 49	Unadjusted OR value (CI 95%)	Adjusted OR^a^ value (CI 95%)
*M1 group*				
mRS ≥ 4 at 90 days	44 (35.2)	30 (61.2)	2.91 (1.47–5.75)	2.81 (1.12–7.02)
mRS ≥ 3 at 90 days	62 (49.6)	35 (71.4)	2.54 (1.25–5.18)	3.56 (1.29–9.84)

Regression model

This table value of quantitative variables expressed as median and interquartile range. Values of qualitative variables expressed as *n* (%)

^a^Final model, adjusted for sex, age, APACHE, NIHSS, GCS and preprocedure mismatch, wake-up stroke, time onset of symptoms to IAMT and orotracheal intubation at ICU admission

## Discussion

There is a close association between hypoxia, neurotoxicity and mortality after a stroke; however, there is poor evidence regarding the possible benefit of oxygen supplementation in non-hypoxemic patients.

Among the few published studies, there is that of Ronning Om et al. [15], a quasi-randomized trial that included a total of 550 patients with ischemic stroke. They randomized two groups comparing oxygen treatment via nasal cannula at 3 L/min during 24 h from the onset of symptomatology, with no routine oxygen. They concluded that there was a lower overall mortality rate per year, and a lower degree of disability at 7 months in the untreated group, although these differences were not significant.

However, Chiu et al. [16] concluded that there was lower mortality (1 vs 6, *p* = 0.048) and a lower incidence of adverse events, specifically pneumonia (1 vs 6, *p* = 0.048), in patients treated with high flow oxygen via mask at 40% compared to administration in nasal cannula at 2 L/min., only for subgroups with complete occlusion of middle cerebral artery.

This study was followed by a larger trial with a similar design, published by Singhal et al. [17], which was terminated early after enrollment of 86 patients, due to an imbalance in deaths favoring control arm (20% vs 8%). These findings are supported by ours, where hyperoxia was associated with a worse functional outcome.

Rincon et al. [18] observed that patients with hyperoxia had higher mortality than those who had normal paO_2_ levels or even with hypoxia (OR 1.7, 95% CI, 1.3–2.1, *p* < 0.001 vs OR 1.3, 95% CI, 1.1–1.7, *p* < 0.010, respectively), in a cohort of 2894 ventilated patients who had suffered a stroke.

However, the results were not as conclusive in the recently published “Stroke Oxygen Study” [19]. A multicenter prospective randomized single-blind trial evaluated routine oxygen treatment during the first 72 h from the cerebrovascular event after randomization to three groups: continuous oxygen therapy with nasal cannula at 2–3 L/min, oxygen therapy only at night for three nights and breathing room air. No significant differences were found in mortality or in the degree of functional dependence reached after 3 months, both by mRS and by Barthel scale. There were several factors that differed significantly from our selected cohort of patients: Only 82% of their cases corresponded to an ischemic stroke, whether in the anterior or posterior location. In addition, their patients did not receive any endovascular mechanical treatment (probably in relation to the low NIHSS at admission, median of 5) and presented a significant delay until randomization (median of 20:43 h from the onset of symptomatology).

Hyperoxia conditions may change depending on the study considered. In the literature, the cutoff from which we consider that there is hyperoxia is not well defined. Evert de Jonge et al. observed that both increased FiO_2_ and high or low paO_2_ during the first 24 h of admission to the ICU proved to be independent factors of mortality, for a sample of more than 36,000 patients. The paO_2_ interval that was associated with lower mortality was 94–123 mmHg [13]. This maximum value of paO2 agrees with that of the patients who obtained a worse functional result in our series. Therefore, we have considered the value of 120 mmHg as the limit to establish the conditions of hyperoxia.

The harmful effects of hyperoxia have been known for decades and are attributed mainly to oxidative stress due to an increase in free radicals [10]. In ischemic stroke, and fundamentally after reoxygenation that occurs during the reperfusion phase, free radicals are generated, which favor the development of an inflammatory response that begins at the microvascular level. This causes an increase in oxidative stress and the reactions that occur in the cytosol and organelles, responsible for causing lesions at the endothelial and parenchymal levels [20]. Some authors have proposed that hyperoxia in critically ill patients could interrupt the establishment of compensation mechanisms (mitochondrial, genes related to HIF-1…), paradoxically causing a detrimental effect [21].

In parallel, in the field of cardiology, there are multiple studies that highlight the deleterious effects of oxygen. It is known to cause constriction in different vascular territories, among which are the coronary arteries, in addition to the cerebral, pulmonary and renal arteries. Thus, ventilation with a high fraction of inspired oxygen is associated with reduced cardiac output [22].

It has been shown that normobaric hyperoxia reduces coronary blood flow by 8–29% in both normal subjects and patients with coronary heart disease or chronic heart failure. This causes a decrease in the release and availability of oxygen at the myocardial level [23].

Based on all the available evidence, the European Society of Cardiology proposed that oxygen should not be routinely administered to patients with suspected acute coronary syndrome unless the oxygen saturation was below 90% [24].

According to these findings, studies in healthy subjects have shown that hyperoxia is associated with a decrease in cerebral blood flow [25], which can be reduced by 11–33% [26, 27].

The reasons why oxygen causes constriction at the microvascular level are still not completely clear. Among other mechanisms is the interruption of compensatory mechanisms, such as the inhibition of prostaglandins, favored by the production of ERO [28], the inactivation of nitric oxide (NO) by superoxide anion [29] or the interruption in the release of ATP by red blood cells in situations of hypoxia [30]. Other studies suggest that vasoconstriction may be related to hyperoxia-induced hypocapnia and not so much related to high oxygen levels [31].

In our series of more than 300 patients, we found an association between admission PaO2 > 120 mmHg and worse functional outcome 90 days after ischemic stroke. Thus, hyperoxia is twice as frequent in patients with mRS ≥ 4 or mRS ≥ 3. This association needs further confirmation by other studies.

It seems necessary to avoid the harmful effects of hyperoxia, in particular for conditions in which an ischemia–reperfusion mechanism prevails, as in the case of stroke [21, 32]. Hyperoxia levels are not well established yet. Thus, and observing our results, it would be prudent to avoid unnecessary administration of FiO2 that raises paO2 levels above the normal range.

This study has several limitations. Thus, being a multidisciplinary team with several services involved, supplemental oxygen until the start of thrombectomy was heterogeneous, especially during pre-hospital care. In addition, it was a prospective study with follow-up without repeated measures, with a single ICU admission blood gas analysis, so it was impossible to know exactly how long the hyperoxia conditions were maintained, since the FiO_2_ provided during the IAMT was arbitrary.

In our series, the thrombectomy procedure was always performed under general anesthesia with intubation. This is a controversial issue, and, in many hospitals, it is performed with the patient awake, in spontaneous breathing. This decision was made in response to the requirements of the Neuroradiology Service to perform all the intra-arterial mechanical thrombectomies in our hospital. We consider that this homogenizes our cohort in terms of the respiratory support administered during the procedure. Perhaps, the results of our study should be applied only in that population of patients to whom IAMT is performed under general sedoanalgesia, intubation and connection to mechanical ventilation.

Despite its limitations, our study shows that, in patients with ischemic stroke undergoing reperfusion therapy, hyperoxia is a variable independently associated with poor functional recovery. This hypothesis should be tested in additional prospective trials.

## Conclusions

There is an association between high oxygen levels in the blood of patients that have suffered ischemic stroke after IAMT and a worse functional outcome established by mRS of 3 scores or more and therefore with a higher degree of dependency.

Based on these results, we cannot assure that hyperoxia worsens the recovery of stroke patients, but it certainly constitutes an independent variable associated with a poor functional recovery. A prospective, randomized study is needed to confirm these results.

Until there is enough evidence that supports routine oxygen supplementation in all stroke patients, we think that its use should be restricted to maintaining oxygen saturation within normal ranges. The purpose of this is to obtain a PaO_2_ level that ensures adequate tissue perfusion. It only seems prudent to avoid high-dose oxygen supplementation that might lead to a state of hyperoxia.

New clinical studies are necessary in order to assess more conservative strategies of oxygen therapy, especially in critical patients with conditions in which there is ischemia–reperfusion, like in the case of stroke.

## Additional files

**Additional file 1.** Appendix that describes in detail the treatment algorithm, the inclusion and exclusion criteria and the anesthetic protocol during intra-arterial thrombectomy.

**Additional file 2.** Appendix that describes in detail the construction of the regression model.

## Data Availability

The datasets used and analyzed during the current study are available from the corresponding author on reasonable request.

## Abbreviations

IAMT: intra-arterial mechanical thrombectomy
ICU: intensive care unit
mRS: modified Rankin scale
rTPA: alteplase
NIHSS: National Institute of Health Stroke Scale Score
GCS: Glasgow Coma Scale
paO_2_: oxygen partial pressure
Mismatch: wearable tissue area
