# Supplementary material for: Association between post-procedural hyperoxia and poor functional outcome after mechanical thrombectomy for ischemic stroke: an observational study
Source: Ann Intensive Care. 2019 May 24;9:59. doi: 10.1186/s13613-019-0533-8 (PMC6534627; doi:10.1186/s13613-019-0533-8)
Supplement: Supplementary file 1 — Additional file 1. Appendix that describes in detail the treatment algorithm, the inclusion and exclusion criteria and the anesthetic protocol during intra-arterial thrombectomy. [file 13613_2019_533_MOESM1_ESM.docx]

Additional file 1. Appendix that describes in detail the treatment algorithm, the inclusion and exclusion criteria and the anesthetic protocol during intraarterial thrombectomy.

**TREATMENT ALGORITHM**

Intravenous fibrinolysis with rtPA will be performed on those patients with ischemic stroke occurring up to 4.5 hours before who meet the established selection criteria *(see inclusion/exclusion criteria)*.

Intra-arterial mechanical thrombectomy will be performed provided that there is large vessel obstruction (intracranial internal carotid artery and trunk of middle cerebral artery) and that any of the following are present, as long as there are no contraindications *(see inclusion/exclusion criteria)*:

a. Ischemic stroke occurring no more than 4.5 hours ago with some contraindications for intravenous fibrinolysis.

b. Ischemic stroke occurring between 4.5 and 8 hours ago, with a mismatch of at least 30%, objectively measured by means of perfusion techniques (CT perfusion and CT angiography).

c. Ischemic stroke occurring no more than 8 hours ago in which intravenous fibrinolysis has failed if large vessel occlusion is still present and there is a mismatch of at least 30% made objectively measured by means of perfusion techniques (CT perfusion and CT angiography): rescue mechanical thrombectomy.

* *The necessary time for in-hospital study in order to decide on thrombolytic therapy shall never exceed 60 minutes.*

* The performance of vascular tests (CT perfusion, CT angiography or arteriography) in order to indicate mechanical thrombectomy shall not delay intravenous fibrinolysis in eligible patients, since the sooner the treatment is performed the greater the benefit for the patient.

** Treatment with rtPA fibrinolysis shall be initiated within the first 90 minutes from onset of symptoms in as many patients as possible. The time elapsed between the rtPA and the rescue thrombectomy shall not exceed, if possible, 90 minutes.*

* In the case of wake-up stroke, thrombolytic treatment will be decided based on imaging criteria (salvageable tissue) instead of time criterion.

**INCLUSION AND EXCLUSION CRITERIA FOR INTRAVENOUS THROMBOLYSIS**

Inclusion criteria:

Patients with acute ischemic stroke occurring no more than 4.5 hours ago for which any of the following exclusion criteria is not applicable.

Exclusion criteria:

1. Intracranial hemorrhage in CT

2. Duration of symptoms > 4.5 hours or ignorance of onset time.

3. Mild symptoms or real improvement before starting the infusion.

4. Acute stroke in accordance with NIHSS clinical criteria > 25 or neuroimaging criteria (hypondensity compatible with infarction >1/3 of middle cerebral artery).

5. Symptoms suggesting subarachnoid hemorrhage although CT is normal.

6. Known bleeding diathesis.

7. Treatment with heparin sodium in the previous 48 hours and elevated aPTT or with LMWH in anticoagulant doses in the previous 12 hours.

8. Oral dicoumarinic anticoagulant therapy. Treatment with rtPA can be considered if INR ≤ 1.7. If the patient is taking direct thrombin inhibitors (dabigatran) or factor Xa inhibitors (rivaroxaban and apixaban) rtPA treatment is not recommended unless specific laboratory tests are normal (thrombin time, ecaring clotting time, factor Xa assay or other tests).

9. Platelet count under 100,000/mm^3^.

10. Glycemia under 50 mg/dL or over 400 mg/dL.

11. Systolic blood pressure > 185 mmHg, diastolic blood pressure > 105 mmHg or need for aggressive measures to control it.

12. Traumatic brain injury or stroke in the previous three months.

13. History of previous intracranial hemorrhage.

14. History of subarachnoid hemorrhage due to ruptured aneurysm.

15. History of lesion in the central nervous system (aneurysm, neoplasia, intracranial or spinal surgery).

16. Hemorrhagic retinopathy.

17. History of cardiac massage, labor or inaccessible blood vessel puncture in the previous 10 days.

18. Ineffective endocarditis, pericarditis.

19. Acute pancreatitis.

20. Gastrointestinal ulcerative disease documented in the previous three months. Esophageal varices. Known intestinal vascular malformations.

21. Neoplasia with increased bleeding risk.

a- Metastatic tumors (relative contraindication): an individual assessment should be made for each case in order to make a decision. It can be absolute if there is:

- Brain metastases or brain primary tumor.

- Large amount of lung metastases (it can be considered after individual assessment, when metastases are few in number (<3), small in size (<3cm), peripheral and without history of bleeding).

- Central lung tumor or metastasis near large vessels.

- Metastasis of choriocarcinoma, melanoma, and of other tumors with large angiogenesis (hemangiopericytoma, angiosarcoma...).

- Life expectancy of less than 6 months.

- History of bleeding caused by a metastatic lesion although it is controlled at present.

b- Rule out the existence of Disseminated Intravascular Coagulation.

c- Active treatment with angiogenesis inhibitors for tumor treatment: bevacizumab, and other oral angiogenesis inhibitors: sorafenib, sunitinib y pazopanib (and other pending commercialization: e.g. regorafenib...). In theory, the treatment with active chemotherapy should not be a contraindication, as long as it meets the aforementioned criteria and the existence of thrombopenia, coagulation alteration, and hemolytic-uremic syndrome is ruled out.

22. Severe liver disease (liver failure, cirrhosis, portal hypertension, active hepatitis).

23. Major surgery or significant trauma in the previous three months.

Considerations:

• Nowadays age > 80 is not considered an exclusion factor for i.v. fibrinolytic therapy. The frequency of favorable functional progress in patients of the SITS registry aged over 80 who were treated with i.v. rtPA was significantly higher than in the case of patients from the neuroprotection trials (VISTA registry) who did not receive thrombolysis, and the effect was similar to the one in groups of younger age (level of evidence 2a).

• Seizures at the beginning of strokes increase the probability of diagnostic error, but it is accepted that they should not be a reason to deny thrombolytic therapy if cerebral infarction is confirmed by neuroimaging techniques.

• The sooner the treatment is administered the greater the benefits of thrombolysis. Therefore, any unnecessary delay must be prevented.

**INCLUSION AND EXCLUSION CRITERIA FOR INTRA-ARTERIAL MECHANICAL THROMBECTOMY**

Inclusion criteria:

1. Age between 18 and 80. Mechanical thrombectomy can be considered in patients >80 years old in some exceptional cases, in patients with excellent quality of life and very low morbidity and mortality (this indication implies specific consent). There are some situations associated with a higher mortality in this age group that can help us individualize patients >80: >82 years old; 3 or + cardiovascular risk factors; woman and atrial fibrillation; lesion in the internal extracranial carotid artery.

2. Ischemic stroke of the trunk of middle cerebral artery (M1-M2 segments) or internal intracranial or extracranial carotid artery within the first 4.5 hours if there is no response to i.v. thrombolysis (rescue mechanical thrombectomy) and within 4.5 and 8 hours after admission.

3. In stroke with acute occlusion of extracranial ICA, it should be considered to start directly with IA therapy if this does not delay treatment (beginning of the same should be < 30 minutes after diagnosis).

4. Wake-up stroke, as long as the results of imaging tests show a mismatch >30% and do not show hypodensity of >1/3 of the middle cerebral artery, age ≤ 80 and <8 hours after the patient’s awakening (this indication shall imply a clarification of personalized risks in the consent).

Exclusion criteria:

1. Intracranial hemorrhage in neuroimaging study.

2. Symptoms suggesting subarachnoid hemorrhage although CT is normal.

3. Mild symptoms or real improvement before starting the infusion. Suspicion of lacunar stroke.

4. Coma or acute stroke with NIHSS > 30.

5. Seizure at the beginning of the stroke, unless imaging proves ischemic stroke.

*6.* Glycemia < 50 mg/dl or > 400 mg/dl.

7. Systolic blood pressure > 185 mmHg or diastolic blood pressure *>*105 mmHg despite intravenous therapy for its control.

8. Existence of bleeding diathesis:

a. Thrombopenia < 30.000 platelets per mm^3^.

b. Heparin sodium in the previous 48 hours and aPTT twice the normal value.

c. Oral anticoagulant treatment with INR > 3. In the case of treatment with a new anticoagulant, until more information is available, the same considerations as with rTPA therapy shall apply.

d. Low molecular weight heparins in anticoagulant doses within the past 12 hours.

e. aPTT >39 seconds, ratio aPTT > 1.5, prothrombin time > 15 seconds, or INR > 1.4-1.7 due to inherited or acquired coagulation disorders.

9. Subacute stroke in CT/MRI taking up >1/3 of the MCA territory or >100 cc of cerebral tissue.

10. No arterial occlusion.

11. Stenosis > 50% proximal to the occluded vessel (relative contraindication, a previous angioplasty can be considered).

12. Suspicion of aortic dissection.

Relative contraindications:

a. Intracranial or spinal surgery, head injury or stroke in other vascular territory in the previous three months.

b. History of intracranial hemorrhage.

c. Terminal illness with little life expectancy or very important comorbidity.

d. Ineffective endocarditis with or without mycotic aneurysms and stroke.

e. Pregnancy (balance the benefits/risks and possibility of radiation protection).

**r-tPA ADMINISTRATION PROTOCOL:**

rtPA administration procedure:

1. Determine inclusion and exclusion criteria.

2. Measure blood pressure (BP) and determine capillary blood glucose. Blood glucose over 400 excludes the patient for treatment. If blood glucose is between 400 and 140 try to decrease blood glucose to levels under 140.

3. Emergency blood test: complete blood count, coagulation factor test and biochemical analysis.

4. Cannulate two peripheral blood vessels, size 18-20G. One of them will be for thrombolytic infusion and the other one will be for maintenance serum therapy.

5. Perform neuroimaging test as soon as possible.

6. Samples shall be delivered to the laboratory with priority by pneumatic tube. The results of the blood tests (except for capillary blood glucose) should not delay rtPA treatment except if there is clinical suspicion of bleeding or thrombocytopenia, if the patient is taking acenocoumarol, warfarin, heparin or a new oral anticoagulant (rivaroxaban, dabigatran, apixaban).

7. Ask for informed written consent from the patient, or an immediate family member if the former cannot give it*.

8. BP should be less than 185/105 mmHg. If it is over 185/105 mmHg in two separate tests with a difference of 15 minutes, pharmacological treatment will be initiated with intravenous antihypertensives. BP should not be under 120/80 mmHg. If these measures do not decrease BP to acceptable levels, the patient will not receive thrombolytic treatment.

9. When the established criteria are met, r-tPA will begin in a dose of 0.9 mg/Kg of weight (maximum 90 mg) in two phases (*Actilyse,* 10, 20, 50 mgr presentations and reconstituted solution 1mgr/ml):

a. 10 % of the total is injected in intravenous bolus in 1 minute.

b. After a waiting period of 3 to 5 minutes, we then proceed to the infusion of the remaining 90% of the dose for 60 minutes, by pump perfusion.

11. Assess neurological function every 15 minutes during infusion. Perform NIHSS an hour after the beginning of the treatment.

12. Assess neurological function frequently for the following hours until 24 hours of treatment. (If possible, every 30 minutes during the first six hours and every hour until the 24 hours after the beginning of infusion have elapsed).

13. If the patient presents with severe headache, nausea, vomiting, decrease in the level of consciousness or worsening in neurological examination, infusion will be stopped and an emergency cranial CT scan will be performed.

14. Measure blood pressure every 15 minutes for the first two hours after initiating fibrinolysis, every 30 minutes for the following 6 hours, and every 60 minutes until 24hours from the beginning of treatment, then according to the usual protocol.

15. Take the necessary measures established in the protocol of hemorrhagic complications in the event of bleeding.

16. Perform control cranial CT scan within 22-36 hours.

17. Monitor the patient at least for 24 hours after treatment.

18. Do not administer aspirin, heparins or oral anticoagulants during the following 24 hours and do not start using them until a control cranial CT scan has been performed ruling out hemorrhagic transformation.

19. The placing of nasogastric or urinary tubes or intra-arterial catheters will be delayed as long as the patient can be handled safely without these devices.

* *Those situations that constitute and emergency and in which not providing a treatment can result in a greater damage than providing it are usually accepted, legally and ethically, as an exception to the need to obtain informed consent. This is applicable when it is impossible to obtain informed consent from the patient and no family member is available.*

**ANESTHETIC MANAGEMENT PROTOCOL IN ACUTE ISCHEMIC STROKE**

It is based on the following objectives:

1. Minimize the necessary time before thrombectomy

a. Efficient anesthetic assessment

b. Minimize delays due to anesthetic procedures

2. Prevent secondary neurological damage

a. Avoid significant hypo- hypertension

b. Avoid hyperthermia, hyperglycemia and hypoxia

3. Treat possible complications:

a. Neurological: convulsions, hemorrhagic transformation and brain edema.

b. Other: airway obstruction, aspiration, arrhythmia, myocardial ischemia, pneumonia, neurogenic pulmonary edema and pulmonary embolism.

1. Sign informed consent.
2. Basic monitoring: electrocardiography, peripheral O2 saturation, non-invasive arterial pressure (NIAP)
3. Place intravenous catheters: 1 or 2 catheters of adequate size preferably in upper limbs.
4. Neurological Monitoring (if adequate): INVOS, BIS.
5. Premedication (if adequate): Midazolam 2mg iv, Atropine 0.5mg iv.
6. Radial artery catheterization (no more than 5 min), if it is not possible, the neuroradiologist will perform femoral artery catheterization with infiltration of local anesthetic.
7. Control of ACT and glycemia at the beginning of procedure. Hourly control of ACT and glycemia.
8. Use of continuous hemodynamic monitoring (if appropriate).
9. Anesthetic induction with invasive arterial pressure (IAP) control:

- Consider rapid sequence and Sellick maneuver.

- Hypnotics (propofol/etomidate, midazolam)

- Muscle relaxants (cisatracurium/rocuronium).

1. Orotracheal intubation instead of placing a laryngeal mask.
2. Bladder catheterization and esophageal temperature probe placement. Hourly control of diuresis and temperature.
3. Central catheterization (if appropriate): internal jugular, subclavian or femoral vein.
4. Maintenance of anesthesia to maintain BIS <60 :

a. Hypnotics:

- Propofol with i.v. perfusion pump.

- Inhalational: Sevorane

b. Muscle relaxants:

- Cisatracurium in perfusion 1 mcg/kg/min

1. Protective mechanical ventilation.
2. Control of BP in order to maintain SBP between 140-180:

a. Hypertension: treat SBP >185 (depth of anesthesia, analgesia, consider antihypertensive: urapidil, esmolol or labetalol as appropriate)

b. Sustained hypotension: treat SBP <120 (excessive depth of anesthesia, adequate fluidotherapy, vasopressors: ephedrine, norepinephrine)

16. Prevent secondary neurological damage:

a. If temperature is ≥38ºC, by physical measures and antipyretic drugs.

b. Prevent glycemia alterations. Treat hyperglycemias >200 mg/dL and hipoglycemias <70 mg/dL.

17. Other measures:

a. Analgesia: Paracetamol 1g iv.

b. Double antiemetic prophylaxis: dexamethasone 4mg i.v. + ondansetron 4mg i.v.

18. Once the neurointerventional procedure is finished, hypnotic drugs and muscle relaxants will stop. Neuromuscular reversal when appropriate.

19. Careful extubation for early neurological assessment and decrease in mechanical ventilation period.

20. Transfer to Intensive Care Unit.

*ACT: Activated clotting time*

*BIS: Bispectral index*

*INVOS: Regional cerebral oxygen saturation index*
