# Supplementary material for: Association between post-procedural hyperoxia and poor functional outcome after mechanical thrombectomy for ischemic stroke: an observational study
Source: Ann Intensive Care. 2019 May 24;9:59. doi: 10.1186/s13613-019-0533-8 (PMC6534627; doi:10.1186/s13613-019-0533-8)
Supplement: Supplementary file 2 — Additional file 2. Appendix that describes in detail the construction of the regression model. [file 13613_2019_533_MOESM2_ESM.docx]

Additional file 2. Appendix that describes in detail the construction of the regression model.

Significant differences in score distribution on the modified Rankin Scale at 90 days after IAMT were observed between the two groups.

Thus, a maximum regression model was obtained, including the confounding variables identified by a previous univariate analysis, whose value of statistical significance was lower than 0.25. Among which were: age, history of smoking, arterial hypertension, diabetes mellitus, COPD, chronic renal failure, previous anticoagulant treatment, baseline rankin score , APACHE score, NIHSS and initial GCS, cataloged as stroke of the awakening, findings in the CT before the TIAM, area of salvable tissue according to missmatch, time to completion of the TIAM, degree of reperfusion achieved and respiratory status at admission in ICU after TIAM, depending on whether they remained intubated orotracheal or not (table 3).

| Table 3. CHARACTERISTICS OF PATIENTS AT ADMISSION IN ICU | | |  |
| --- | --- | --- | --- |
| Characteristic | **mRS<4**  **N = 169** | **mRS ≥4**  **N = 164** | **Significance level**  **(p value)** |
| Age in years | 67 (55-76) | 74 (66-79) | <0.01 |
| Male sex | 84 (49.7) | 78 (47.6) | 0.70 |
| Body mass index | 27 (24-29) | 27 (25-30) | 0.32 |
| History of smoking | 57 (33.7) | 40 (24.4) | 0.06 |
| Baseline Rankin   - 0 - 1 - ≥2 | 130 (76.9)  27 (16.0)  12 (7.1) | 107 (65.6)  36 (22.1)  20 (12.3) | 0.02  0.02  0.16  0.11 |
| Hypertension | 104 (61.5) | 121 (73.8) | 0.02 |
| Dyslipidemia | 71 (42.0) | 73 (44.5) | 0.64 |
| Diabetes mellitus | 40 (23.7) | 55 (33.5) | 0.05 |
| History of COPD | 10 (5.9) | 16 (9.8) | 0.19 |
| History of CRF^a^ | 7 (4.1) | 12 (7.3) | 0.21 |
| Atrial fibrillation/ flutter | 65 (38.5) | 68 (41.5) | 0.58 |
| Previous stroke | 22 (13.0) | 16 (9.8) | 0.35 |
| Previous antiagregating therapy | 49 (29.0) | 54 (32.9) | 0.44 |
| Previous anticoagulation therapy | 22 (13.0) | 31 (18.9) | 0.14 |
| Preprocedure GCS | 15 (14-15) | 14 (11-15) | <0.01 |
| Preprocedure NIHSS | 16 (11-20) | 19 (16-22) | <0.01 |
| APACHE | 13 (12-17) | 18 (14-24) | <0.01 |
| Findings in TC   - Without ischemia - Acute ischemic areas^b^ - Areas of ancient stroke | 74 (44.3)  73 (43.7)  20 (12.0) | 59 (36.4)  89 (54.9)  14 (8.6) | 0.12  0.14  0.04  0.32 |
| Mismatch | 70 (60-80) | 60 (40-80) | <0.01 |
| Etiology of stroke   - Cardioembolic - Atherothrombotic - Idiopathic | 64 (37.9)  61 (36.1)  44 (26.0) | 66 (40.2)  56 (34.1)  42 (25.6) | 0.90  0.66  0.71  0.93 |
| Wake-up stroke | 14 (8.3) | 23 (14.0) | 0.10 |
| Time (min.) onset of symptoms to IAMT | 270 (210-360) | 290 (211-390) | 0.17 |
| Type of intervention   - Thrombectomy - Thrombectomy+ stent | 144 (85.2)  25 (14.8) | 137 (83.5)  27 (16.5) | 0.67 |
| Reperfusion degree^c^   - Successful reperfusion - Non-successful reperfusion | 164 (97.0)  5 (3.0) | 144 (87.8)  20 (12.2) | <0.01 |
| OTI at admission^d^ | 71 (42.0) | 105 (64.0) | <0.01 |

Table 3.Values of quantitative variables expressed as median and interquartile range. Values of qualitative variables expressed as n (%).

^a.^. Creatinine clearance < 60 ml/min/1.73 m2

^b^. Included signs of acute ischemia and indirect signs of ischemia: hyperdense middle cerebral artery sign, hemispheric sulcus and/or insular cortex effacement, contrast attenuation, ventricular compression.

^c^. Successful reperfusion included TICI 2b and TICI 3. Non-successful reperfusion include TICI≤2a

^d^. Patients intubated at admission in ICU after IAMT

Finally, the maximum model was simplified according to the Maldonado and Greenland’s criteria. It was adjusted only for those variables whose exclusion from the model involved a modification of the OR of the hyperoxia variable greater than 10%, according to the parsimony principle: APACHE score, area of salvageable tissue, and persistence of orotracheal intubation upon admission in ICU.

In addition, we considered that sex should be included, despite not inducing confusion, given the existence of scientific arguments in the literature that establish differences between the sexes in terms of the prognosis of stroke ^1^, the degree of reperfusion achieved after TIAM, as well as such as the NIHSS score prior to thrombectomy, due to its importance when interpreting the results. No interaction variables were identified in the model.

| **MAXIMUM MODEL** | |  | | | | |
| --- | --- | --- | --- | --- | --- | --- |
|  | | | **Regression coefficient** | **Significance level (p value)** | **OR (IC 95%)** |  |
|  | **pO_2_ > 120 mmHg** | | 0.80 | 0.02 | 2.22 (1.15-4.29) |  |
|  | **Sex** | | 0.03 | 0.92 | 1.04 (0.54-1.98) |  |
|  | **History of smoking** | | 0.26 | 0.51 | 1.29 (0.59-2.81) |  |
|  | **Age** | | 0.03 | 0,07 | 1,03 (1.00-1.06) |  |
|  | **Diabetes mellitus** | | -0.08 | 0.82 | 0.92 (0.45-1.88) |  |
|  | **OTI at admission^a^** | | 0.14 | 0.68 | 1.15 (0.59-2.21) |  |
|  | **Hypertension** | | -0.15 | 0.69 | 0.86 (.042-1.77) |  |
|  | **History of COPD^b^** | | 0.19 | 0.74 | 1.20 (0.40-3.63) |  |
|  | **History of CRF^c^** | | -0.78 | 0.26 | 0.46 (0.12-1.76) |  |
|  | **Previous anticoagulation therapy** | | -0.79 | 0.06 | 0.45 (0.20-1.05) |  |
|  | **Wake-up stroke** | | -0.70 | 0.17 | 0.49 (0.18-1.37) |  |
|  | **Baseline Rankin** | | -0.06 | 0.82 | 0.94 (0.57-1.56) |  |
|  | **APACHE** | | 0.16 | <0.01 | 1.18 (1.10-1.26) |  |
|  | **Preprocedure NIHSS** | | 0.07 | 0.06 | 1.07 (1.00-1.15) |  |
|  | **Preprocedure GCS** | | <0.01 | 0.94 | 1.00 (0.85-1.19) |  |
|  | **Findings in CT scan** | | 0.08 | 0.65 | 1.08 (0.78-1.50) |  |
|  | **Mismatch** | | -0.02 | <0.01 | 0.98 (0.96-0.99) |  |
|  | **Time (min.)onset of symptoms -IAMT** | | <0.01 | 0.08 | 1.00 (1.00-1.00) |  |
|  | **Reperfusion degree** | | 0.32 | 0.63 | 1.37 (0.37-5.06) |  |
|  |  | |  |  |  |  |
|  | | | | | | |

*^a.^. Patients intubated at admission in ICU after IAMT*

*^b.^. Chronic obstructive pulmonary disease*

*^c^. Creatinine clearance < 60 ml/min/1.73 m2*

| **FINAL MODEL** | |  | | | | |
| --- | --- | --- | --- | --- | --- | --- |
|  | | | **Regression coefficient** | **Significance level (p value)** | **OR (IC 95%)** |  |
|  | **pO_2_ > 120 mmHg** | | 0,82 | 0,01 | 2,27 (1.22-4.23) |  |
|  | **Sex** | | -0,11 | 0,70 | 0,89 (0.50-1.58) |  |
|  | **Age** | | 0,03 | 0,02 | 1,03 (1.00-1.06) |  |
|  | **OTI at admission^a^** | | <0,01 | 0,99 | 1,00 (0.54-1.84) |  |
|  | **APACHE** | | 0,15 | <0.01 | 1,16 (1.09-1.23) |  |
|  | **Preprocedure NIHSS** | | 0,08 | 0,01 | 1,08 (1.02-1.15) |  |
|  | **Mismatch** | | -0,02 | <0.01 | 0,98 (0.96-0.99) |  |
|  | **Reperfusion degree** | | 0,35 | 0,56 | 1,42 (0.43-4.69) |  |
|  |  | |  |  |  |  |
| ^a^. *Patients intubated at admission in ICU after IAMT* | | | | | | |

The correct calibration of the model was verified based on the Hosmer-Lemeshow test, p= 0.47.

The model obtained showed a sensitivity of 74.6% and a specificity of 71.3%.

P**redictive capacity of the model**

| **Observed** | | **Predicted** | | |  |
| --- | --- | --- | --- | --- | --- |
|  |  | **mRS ≥4** | | |  |
|  |  | **No** | **Yes** | **Total** |  |
| **mRS ≥4** | **No** | 106 | 36 | 142  136 | Sens = 74.6  Spec = 71.3 |
|  | **Yes** | 39 | 97 |  |  |
| **Total** | | 145 | 133 | 178 |  |

*^1^. Sensibility. Expressed value %.*

*^2^. Specificity. Expressed value %.*

The discriminative capacity of the model was evaluated through the construction of a ROC curve. An area under the curve of 0.81 was obtained.

| **Area under the curve** | | | | |
| --- | --- | --- | --- | --- |
| **AUC** | **Standard error** | **Asymptotic significance** | **Asymptotic confidence interval (95%)** | |
|  |  |  | **Lower limit** | **Upper limit** |
| 0,81 | 0,02 | <0.01 | 0,76 | 0,86 |

A subgroup analysis was performed according to the stroke lesion location. The confounding variables identified in the univariate analysis are shown in Table 4. A logistic regression adjusted to these variables was performed.

| Table 4. M1 GROUP. CONFOUNDING VARIABLES INCLUDED IN THE REGRESSION MODEL | | |  |
| --- | --- | --- | --- |
| Characteristic | **mRS<4**  **N = 100** | **mRS ≥4**  **N = 74** | **Significance level**  **(p value)** |
| Age in years | 71 (56-78) | 74 (64-78) | 0.07 |
| Male sex | 49 (49.0) | 35 (47.3) | 0.82 |
| Preprocedure GCS | 15 (14-15) | 14 (11-15) | <0.01 |
| Preprocedure NIHSS | 16 (10-20) | 18 (16-22) | <0.01 |
| APACHE | 13 (10-17) | 18 (15-22) | <0.01 |
| Mismatch | 70 (65-85) | 70 (50-80) | 0.11 |
| Wake-up stroke | 8 (8.0) | 12 (16.2) | 0.09 |
| Time (min.) onset of symptoms to IAMT | 261 (206-330) | 315 (230-392) | 0.02 |
| OTI at admission^a^ | 39 (39.0) | 49 (66.2) | <0.01 |

Table 4.Values of quantitative variables expressed as median and interquartile range. Values of qualitative variables expressed as n (%).

^a.^. Patients intubated at ICU admission after IAMT

1. Appelros, P., Stegmayr, B. & Terent, A. Sex differences in stroke epidemiology: A systematic review. *Stroke* **40,** 1082–1090 (2009).
